# In Silico Prediction of Large-Scale Microbial Production Performance: Constraints for Getting Proper Data-Driven Models

**DOI:** 10.1016/j.csbj.2018.06.002

**Published:** 2018-07-06

**Authors:** Julia Zieringer, Ralf Takors

**Affiliations:** Institute of Biochemical Engineering, University of Stuttgart, Germany

**Keywords:** Gene regulatory networks, Scale-down devices, CFD, Compartment models, CFD-based compartment models

## Abstract

Industrial bioreactors range from 10.000 to 700.000 L and characteristically show different zones of substrate availabilities, dissolved gas concentrations and pH values reflecting physical, technical and economic constraints of scale-up. Microbial producers are fluctuating inside the bioreactors thereby experiencing frequently changing micro-environmental conditions. The external stimuli induce responses on microbial metabolism and on transcriptional regulation programs. Both may deteriorate the expected microbial production performance in large scale compared to expectations deduced from ideal, well-mixed lab-scale conditions. Accordingly, predictive tools are needed to quantify large-scale impacts considering bioreactor heterogeneities. The review shows that the time is right to combine simulations of microbial kinetics with calculations of large-scale environmental conditions to predict the bioreactor performance. Accordingly, basic experimental procedures and computational tools are presented to derive proper microbial models and hydrodynamic conditions, and to link both for bioreactor modeling. Particular emphasis is laid on the identification of gene regulatory networks as the implementation of such models will surely gain momentum in future studies.

## 1 Introduction

With the advent of metabolic engineering in the 1990s [1], the engineers' view on microbes changed. Process optimization no longer considered the extracellular environment (i.e. cultivation conditions) alone, but started to investigate intracellular mechanisms in addition [1, 2]. Since then, intracellular reaction rates have been quantified and models of regulatory processes finally aiming at identifying targets for further strain and process improvement have been derived. To some extent driven by the observations that cellular engineering always results in multiple and complex systemic responses [1], furthermore catalyzed by the avalanche of omics data that were accessible, systems biology and systems metabolic engineering emerged in 2000. In essence, holistic models have been developed that aim to provide as sound and comprehensive a cellular view as possible.

The development clearly reflects the general engineering mindset of investigating the whole system by modularization, quantitative analysis, reassembling and studying the interaction of the networked modules. The earliest, simple examples may be given by the Monod growth model [3], followed by more sophisticated approaches like the lactose operon considering feedback regulation in *Escherichia coli*, finally leading to complex models comprising multiple levels of cellular regulation [4]. While such movements led to the birth of systems biology [5] and systems metabolic engineering [[6], [7], [8], [9]] core engineering activities such as scale-up were a matter of steady development, too.

Scale-up is the procedure to transfer lab-bioprocesses in production (large) conditions, often covering 7 to 8 orders of magnitude of volume. Unfortunately, loss or even failure of large-scale performance may occur. Detailed knowhow is necessary to prevent unwanted production losses. Accordingly, Oosterhuis and Kossen were the first who presented a scale-up simulator (1983) for investigating the impact of oxygen gradients on *Gluconobacter oxydans* [10]. They further introduced bioreactor compartment models to achieve the coarse spatial resolution of local oxygen transfer rates to identify micro- and anaerobic zones [11]. This line of thinking was followed by a series of similar studies [[12], [13], [14], [15], [16]] and reached a new level of complexity by linking simulations of hydrodynamics and mass transports with simple metabolic models of *Saccaromyces cerevisiae* and *E. coli* [[17], [18], [19], [20], [21]]. Notably, cellular dynamics were modeled by focusing on metabolism dynamics only. This is remarkable as systems biology has already shown that holistic models are able to cover a far broader range of complexity. Scale-up engineers have already pointed out [22] that profound knowhow is necessary to enable the best knowledge-based scale-up using in silico predictions.

This review addresses the current need for knowledge-based process scale-up by elucidating the putative contributions of modeling. The existing plethora of modeling approaches will be structured with respect to granularity and usefulness to (i) identify and (ii) model key regulatory phenomena and (iii) to link cellular models with predictions of large-scale hydrodynamics. It will be shown that the time is right to approach the challenging goal of in silico predicted large-scale performance of microbial producers.

## 2 Data-driven Approach

Comprehensive data sets are necessary to develop gene regulatory models, generated to answer the biological question of interest. This also holds true for elucidating complex metabolic and regulatory responses of producer cells that are exposed to industrial production conditions. One approach to collect representative data is to mimic large-scale conditions and to capture time series of regulatory dynamics as a basis for unraveling dynamic regulatory models. Such approaches usually require rapid sampling experiments that ‘freeze’ metabolic states monitored in scale-down experiments. Examples of experimental procedures are given in the following.

### 2.1 Experimental Set-Ups Mimicking Large-Scale Heterogeneities

**Fig. 1:**
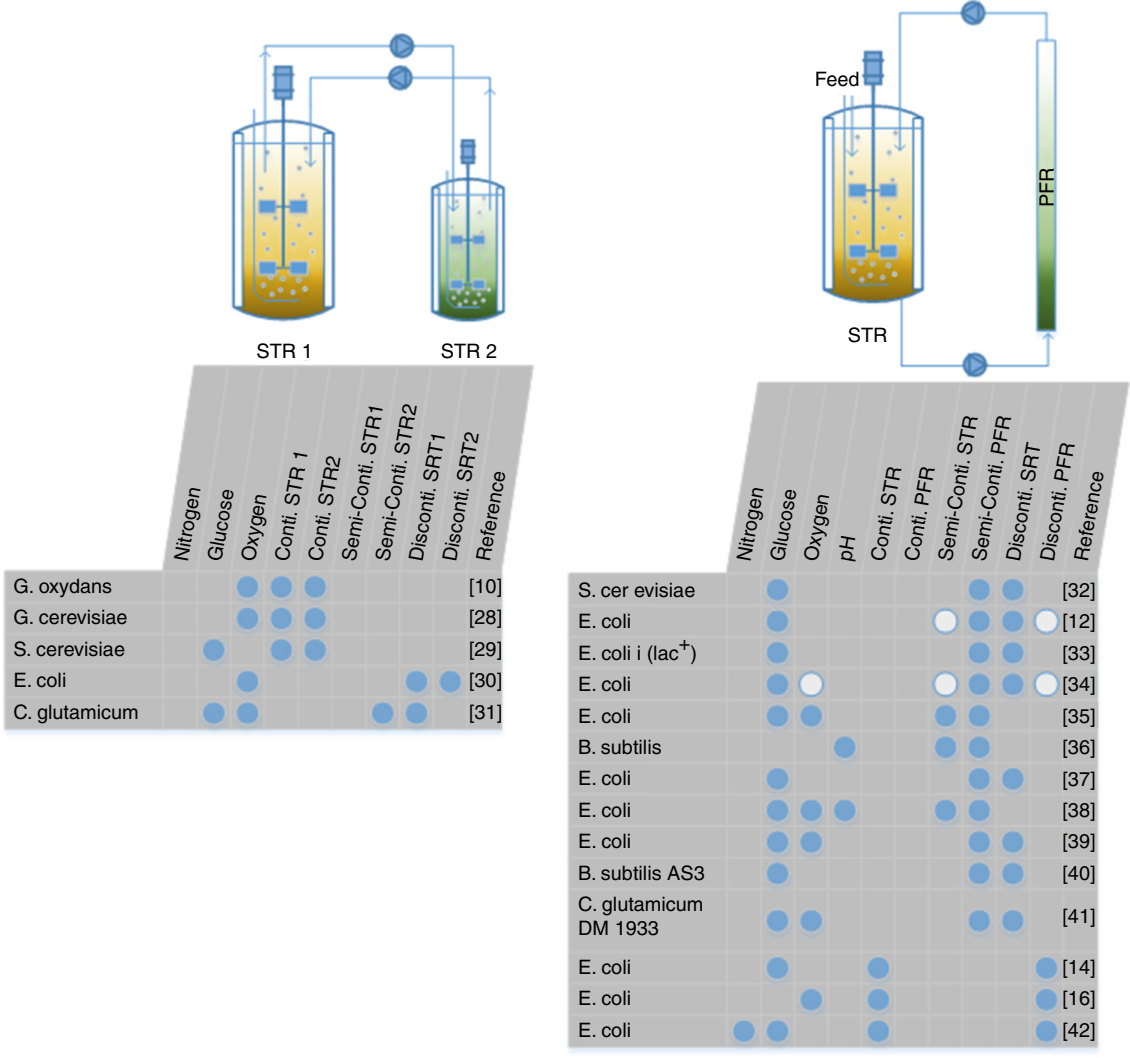
Matrix of STR-STR and STR-PFR applications with different fluctuating conditions and operation modes (blue dots). The *E. coli* strain is the standard strain W3110. Alternative approaches or different operation modes within the same publication are displayed as blue circles with a white filling. The experimental setups are arranged by the year of publication. Investigations with redundant application information are mentioned once according to the most recent paper.

In large-scale production processes micro-environmental inhomogeneities often occur. Insufficient mixing leads to severe axial and horizontal concentration gradients. Producer cells frequently cross these poorly mixed zones which triggers metabolic and transcriptional responses accordingly [23]. Because large-scale experimental data are rarely accessible, experimental scale-up simulators are typically applied, reflecting large-scale conditions [24]. Pioneering studies were performed by Oosterhuis et al. [10] using a two compartment system comprising two stirred tank reactors (STRs) to investigate the effect of different oxygen levels upon the gluconic acid fermentation of *Gluconobacter oxydans*. Since then, variations of the two compartment set up considered the combination of an STR and a plug flow reactor (PFR). Reviews have been given by Delvigne et al. and Neubauer and Junne [22, 25, 26]. Fig. 1 depicts selected examples for several STR-STR and STR-PFR applications.

Experimental scale-up simulators do not merely consist of two compartments. Three compartment approaches have been studied as well. Examples are the STR-STR-STR cascade of Buchholz et al. [13] and the PFR-STR-PFR set-up of Lemoine et al. [28]. Accordingly, more complex scale-up scenarios could be analyzed.

Notably, two and three compartment scale-up simulators mirror the cellular responses on repeated, frequent stimuli. In contrast, investigations of single perturbations may be a proper tool for deriving distinct stimulus/response correlations, see Fig. 1 for examples. On this basis, explicit metabolic and transcriptional dynamics can be deduced that, when properly superimposed, result in the complex cellular response observed. However, signal transduction is highly networked in the cells which may cause the cross-interference of multiple stimuli. The coincidence of multiple stimuli in large-scale fermentation is the rule rather than the exception [29, 30]. Accordingly, multiple stimulus/response studies are likely to gain importance in the future.

### 2.2 Experimental Access to Metabolic and Transcriptional Responses

Samples taken from the scale-up simulators need to be processed so that metabolic and transcriptional states are ‘frozen’ immediately. Metabolic inactivation and purification can be achieved via several approaches [[31], [32], [33], [34]] and requires individual optimization for the given problem. Blocking intracellular transcription is achieved by sampling into RNA protect kits [14]. Correctly prepared, samples can be treated further to identify metabolic compositions via metabolic profiling or fingerprinting techniques [[35], [36], [37]], protein contents via affinity tags [38] or mass spectrometry [39] and transcript levels, either applying microarrays or, more preferred, next generation sequencing technologies analyzing mRNAs [[40], [41], [42]]. To reduce the overall sequencing expenses, library preparation usually is done via a rRNA depletion or poly-A enrichment step to remove non-coding rRNA.

**Fig. 2:**
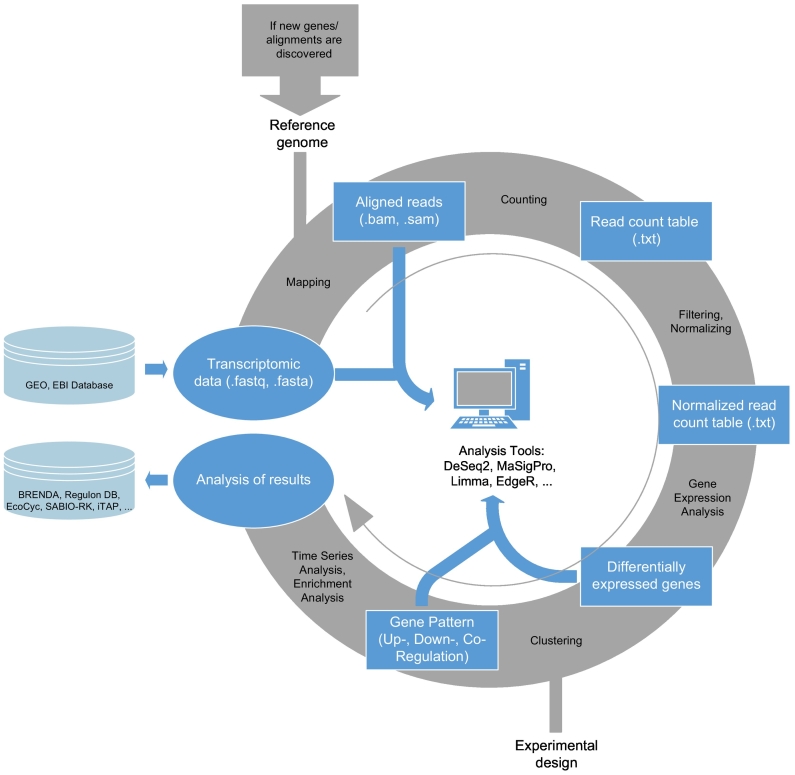
Workflow illustrating the general procedure when analyzing gene expression data. RNA Seq analysis creates FASTA and FASTQ as raw data formats compiled in gene expression repositories (GEO, EBI), followed by SAM or BAM files for aligned reads. The analysis steps are, in general: (1) Mapping the reference genome onto the transcriptomic data, (2) Counting reads, (3) Filtering low read counts and normalizing counts, (4) Gene Expression Analysis, (5) Clustering, (6) Time Series Analysis and Enrichment Analysis. DeSeq2, MaSigPro, limma and edgeR are often applied packages within the language R to analyze transcriptomic data, in case of differential gene expression as well as gene pattern analysis. The resulting dynamic expressions and parameters are stored in databases like BRENDA [44], Regulon DB [45], EcoCyc [46], SABIO-RK [47] and iTAP [48].

Various methods for RNA Seq analysis are available and have been reviewed recently by Conesa et al. [43]. Regarding modeling, time series of transcripts are particularly important which requires methods of differential gene expression analysis. Fig. 2 provides an overview of a typical workflow making use of public R packages.

Once time series of transcripts are available, modelers may be interested in unraveling gene clusters showing similar transcription dynamics and data integration in dynamic models. Applicants may be guided via evaluating reports of Rapaport et al. [49], Hecker et al. and Banf et al. [50, 51]. Currently, algorithms such as DeSeq2 [52] and MaSigPro [53] are often applied.

Application examples are given by transcript time series and monitoring of metabolic changes reflecting the stimuli of glucose [14, 15, 54], nitrogen [55], oxygen [16, 56] or temperature stress [57] of *E. coli*. Data like this, derived from transcriptome analysis as it is described in Fig. 2, are the basis of proper validated mathematical models. Transcript analysis even enabled the engineering of robust *E. coli* strains [58] by attenuating the level of the alarmone ppGpp, the inducer of the stringent response regulation program. The new host is able to maintain high glucose uptake rates even under non or slow growing conditions.

### 2.3 Experimental Access to Single Cell Analysis

It is a well known fact that microbial populations in bioreactors are rather heterogeneous than homogeneous. A combination of stimuli such as local substrate availabilities, temperature and pH conditions may induce differences in cell cycle status, cell division, growth rates, etc. [59].

Such subpopulations can be experimentally analyzed via studies, for instance using fully-automated real-time, flow injection flow cytometry (FI-FCM) [60, 61] or real-time imaging in combination with microfluidic cultivation devices [[62], [63], [64]].

Bennett and Hasty [65] reviewed several microfluidic devices which can be used to examine intracellular signaling pathways and the dynamics of gene regulation in bacteria, yeast and higher eukaryotes on a single cell basis. Often, on-line monitoring is combined with microfluidic studies to achieve full resolution of complex interactions. These technologies are expected to yield novel insights and allow the construction of mathematical models that more accurately describe the complex dynamics of gene regulation [65]. Lemoine et al. provided a review about tools for monitoring population heterogeneities on a single cell basis [66]. Today, even single cell transcription analysis using novel sequencing technologies is becoming achievable [67] which may further increase the quality of mathematical models.

## 3 Modeling Microbial Growth With Different Granularity

Based on proper analyzed experimentally data sets, mathematical models can be derived to simulate the microbial behaviour under different conditions with a varying level of detail. Following the well-known classification of Bailey [68] microbial models can be divided into non-structured/structured and non-segregated/segregated approaches. Non-structured/Non-segregated approaches represent the simplest growth models assuming average cells without subcellular detail. Such models are typically applied for bioprocess design. For the sake of simplicity, they are also applied in agent-based modeling for tracking individual cells. The consideration of subpopulations or individual cell properties leads to segregated approaches which, thanks to the improving availability of experimental data, is gaining more and more attraction. Structured, non-segregated models are commonly used for implementing the subcellular details of metabolic and transcriptional regulation, compartmentation or signal transduction [69, 70]. They are computationally intensive but represent a powerful tool for predicting detailed cellular responses to extracellular stimuli. The most accurate approach are structured/segregated models [71], which for example describe the whole glycolysis process with reactions for each enzyme, depending on enzyme affinites and turn over rates. These paramters are more difficult to identify but transferable to other conditions. However, models like this are limited in scale, due to the complexity of the cellular mechanisms and the single cell consideration, which results in a quadratic scaling problem.

### 3.1 Identifying Structured and Non-Structured Microbial Models

Non-segregated, structured models typically consist of a rigid network structure and a set of rate expressions including sensitive parameters. Knowledge of the network structure, the kinetic equations and the parameters is key to identifying a proper model. Often, such structures are determined following the bottom-up approach, i.e. the statistically profound identification of correlations between the structuring elements based on experimental data. The bottom-up concept can be applied to merge already existing small-scale models into large models [[72], [73], [74]]. Alternatively, top-down approaches aim for the identification of model parameters for a given structure. Accordingly, the top-down approach is a powerful tool for deciphering details of pathway interaction with the network, provided that the given structure is correct [75, 76]. Table 1 depicts a comparison of the two approaches, including prediction goals and limitations. Statements hold true irrespective of whether model complexity is limited to metabolic interactions or whether superior regulation levels such as transcriptional or post-translational feedbacks are included.

**Table 1:** Comparison of bottom-up and top-down approach.

	Bottom-up	Top-down
Design steps	From single molecule to pattern	From single elements to relations
Model size	Small-scale	Coarse-grained large-scale
Model complexity	Detailed	Global
Prediction goals	Detailed time-scale resolution	Global cellular dynamics
Limitations	Lack of kinetic parameters and in-depth knowledge	Neglect of single reaction steps

### 3.2 Identifying Gene Regulatory Networks (GRNs)

When cells are exposed to dynamic stimuli, such as the fluctuating micro-environmental changes in large-scale bioreactors, they show short- and long-term physiological responses. Whereas the first are dominated by metabolic interactions, the second include strategies for microbial adaptation usually comprising changes of transcriptome and proteome. However, recent findings [[14], [15], [16], 54] have shown that transcriptional response occurs massively, even during short-term, sub-minute periods. Accordingly, GRN models gain importance even for modeling short-term responses which explicitly motivates their use.

A gene regulatory network links transcription factors to their target genes thereby creating a dynamic interaction map connecting external stimuli with internal transcriptional and even metabolic responses. Accordingly, GRN models may comprise the signal stimulus, its transduction to the receptor, the transcriptional response and downstream processes such as translation, post-translational modifications of protein and protein degradation. Altogether, these interactions form a very complex regulatory network. Roughly, the plentitude of GRNs may be divided into three representative approaches: continuous models (in this case based on ordinary differential equations) [71, [86], [87], [88], [89], [90], [91]], Boolean models [[92], [93], [94], [95]] and probabilistic models [[96], [97], [98], [99]]. These and other methods, such as Petri nets, Bayesian networks or neural networks, have been extensively reviewed by Karlebach and Shamir [100] and Machado et al. [101].

Stochastic models start from the assumption that gene expression should be described by random events e.g. caused by the shortage of mRNA molecules and factors of transcription. Similarly, initiation and elongation factors are scarce and may cause stochastic translation processes. Accordingly, the continuum paradigm, i.e. the assumption of a sufficient, homogeneous availability of each model entity, may be questionable and could be checked using the chemical master equation. In case the number of molecules per cell is too low, stochastic models should be considered [102]. Pragmatic guidelines have been published by Kremling et al. [103] and Turner et al. [97], identifying molecule numbers of about 100 per cell as the threshold value.

Alternatively, systems of ordinary differential equations (ODEs) can be applied ignoring stochastic transcription events and assuming cellular continuum instead. Cellular entities are simulated as continuous time courses. Such models require knowledge about gene regulatory mechanisms to select appropriate rate laws and to identify corresponding rate parameters for parameter estimation. Standardized formats simplify the development process and encourage the automatic construction of kinetic models. However, the prediction quality of the model may deteriorate for the prediction of cellular states that are not reflected by the experimental data. Checking thermodynamic feasibility for estimated fluxes is strongly encouraged to prevent misleading findings [104]. If only a small amount of data is available, Boolean approaches may be useful to model regulatory networks. As a key feature, Boolean models consider *on/off* activation and the inhibition of transcription factors and genes. Accordingly, such models are helpful to predict *on/off*-like gene switching but fail to simulate distinct time series of transcriptional dynamics that occur after frequent stimulations in large-scale bioreactors.

Moreover, Mochizuki [105] showed that high prediction qualities and the easy handling of Boolean models is limited to small-scale models. Consequently, large-scale GRNs should preferably be composed of ODEs [100]. A short summary of the different class of models with their specific advantages and disadvantages is given in Table 2.

**Table 2:** Summary of advantages, disadvantages and application of the above mentioned methods.

Model class	Application	Advantages	Disadvantages
Structured	Systems in transient state	Cellular compartmentation	Biological knowledge
Non-structured	Steady-state systems	Easy to build	Only phenomenological cell description
Segregated	Heterogeneous, individual cell systems	More representative and informative	Difficult to handle mathematically
Non-segregated	Systems with average cell description	Easy to build, for a large number of cells	Average cell description
Probabilistic	Randomly distributed events in time	Realistic behaviour	Only for low number of molecules in cell
Continuous	Evenly distributed events in time (cellular continuum)	Large-scale possible	Detailed knowledge about mechanisms and biological parameters
Boolean	Discrete dynamical system	Small amount of experimental data (On/Off conditions)	Fails to simulate distinct time series

Inferring gene regulatory networks from gene expression data remains a challenging task due to the large number of potential interactions, the relatively small number of available measurements and the intrinsic noise often caused by the biological variance which reflects the heterogeneity of the cell population. Despite success with automated model set-up and identification, manual curation of the inferred network interactions can become time intensive and cumbersome due to the amount of data investigated [106].

To achieve high prediction quality, kinetic parameters need to be accurate and sensitive, i.e. parameter variance should be low and parameter sensitivity should be high to enable highly accurate model prediction with the lowest amount of parameters necessary. Parameter values may be extracted from experimental data, taken from public databases or already existing dynamic models and kinetics used.

Parameters of the GRN model can be deduced from experimental data usually applying least-square error estimation. In essence, parameter estimation is an optimization problem which minimizes the weighted squared distance between simulation and experimental observation to achieve a parameter set for the least squares. Such approaches can be combined with model discrimination methods [107]. In case the parameter estimation problem does not have a unique solution, the solution space can be further constrained using thermodynamic laws or by expanding the experimental basis taking into account other experimental conditions to challenge the applicability of the model [108]. Parameter estimation methods have been reviewed by Lillacci and Khammash [109].

For example, small-scale regulatory networks of *E. coli* [71, 87, 110] and also large or genome-scale regulatory networks [98, [111], [112], [113]] have already been published. In general, applicants should pay attention to the transferability of the models because reference conditions may be different compared to the current case which is likely to cause the improper extrapolation of experimental findings.

Once a model has been identified, its validity needs to be checked against new data sets that were not used for parameter identification. When models can successfully simulate such new data, it is a strong indication that the mechanistic principles and assumptions behind the model are sound. If a model fails to pass the validation step, the modeler needs to revise the previous steps of their modeling process.

Recent examples of data-driven GRN models are given by Erickson et al. [76], Palsson and Nielson [[114], [115], [116], [117]] and others [98, [118], [119], [120], [121], [122]]. For example, Klipp et al. [72, 123] describes bacterial growth transitions considering the proteome level, the complex interactions of the yeast cell cycle and the prediction of complex regulatory patterns following the mindset of optimized resource allocation in yeasts, respectively. The current developments of metabolism and gene expression (ME) models are in line with the pioneering approach of Chassagnole et al. [71] who published a comprehensive dynamic model of the central metabolism in *E. coli*.

## 4 Simulating the Cellular Environment With Embedded Growing Cells

Large-scale bioreactor conditions need to be calculated, aiming at a spatial resolution of the mass, momentum and energy balances via numerical simulation. In particular, the Navier-Stokes equations (NSE) representing the conservation of momentum, the continuity equation representing the conservation of mass and the energy equation predicting the temperature in the fluid of a multiphase system have to be considered. The Navier-Stokes equations basically describe the motion of viscous fluid flows where the fluids are considered as a continuum rather than a number of colliding particles. Under the typical mixing conditions given, the occurrence of turbulent zones is likely. Turbulence is defined as a state consisting of structures such as eddies which affect molecular diffusion, heat transfer and the mixing behavior.

### 4.1 Modeling of Hydrodynamics and Mass Transfer

**Fig. 3:**
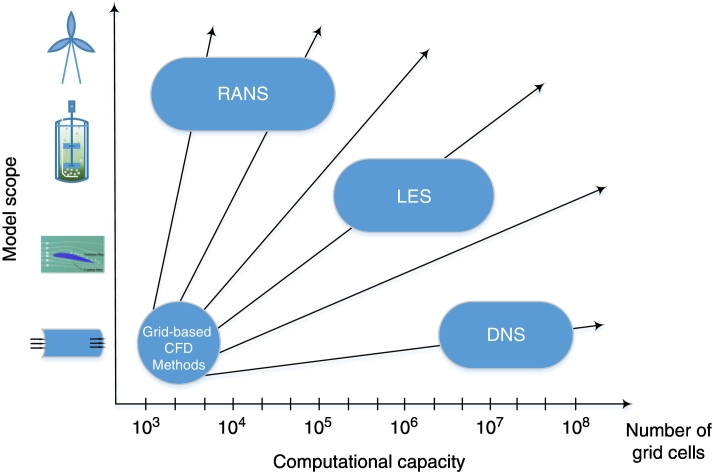
Different approaches of turbulence models, regarding the model scope and fields of application, as well as the corresponding computational capacity required. RANS: Reynolds-averaged Navier-Stokes equation, LES: Large Eddy Simulation, DNS: Direct Numerical Simulation.

To describe hydrodynamic turbulence, multiple suggestions have been published, often applying the modified Reynolds-averaged Navier-Stokes equation (RANS) for multiphase systems [124]. Other alternative approaches, such as Large Eddy Simulation (LES) and Direct Numerical Simulation (DNS), offer increased accuracy, but require immense computational capacity [125, 126] as displayed in Fig. 3.

The Reynolds-averaged Navier-Stokes equations are time-averaged equations of motion for turbulent flows approximating different turbulent scales through fluctuating quantities, an idea first proposed by Reynolds [127]. RANS models offer the most economic approach for simulating complex turbulent flows, because turbulences are considered with different levels of complexity. The most common RANS turbulence models are classified with respect to the number of additional transport equations that need to be solved along with the RANS flow equation. Besides, the often used two-equation models, such as the standard k-*ϵ*, k-*ω* or Renormalization group k-*ϵ* models, one-equation models (low-cost RANS models, e.g. the Spalart-Allmaras approach) or even zero-equation models which estimate the turbulence viscosity via the mean velocity and the length scale using an empirical formula are available [128]. Details are given in the review of Rodi [129].

In addition to the simulation of turbulence, the proper modeling of interactions between different phases (e.g. aqueous media, air bubbles and solid cells) is a challenge. Table 3 provides an overview of three common approaches.

**Table 3:** Comparison of CM and CFD model approaches.

	CFD		
	Euler-Euler	Euler-Lagrange	Compartmentation
Prediction purpose	Reactor heterogeneity	Cell-Environment Interaction	Reactor design
Computational effort	High	High	Low
Level of detail	High	High	Low
Prediction accuracy of flow regime	High	High	Low
Single cell tracking	No	Yes	Yes
Integrable model size	Coarse-grained small-scale	Coarse-grained small-scale	Genome-scale
Amount of particles	High	Low (<10%)	High

**Fig. 4:**
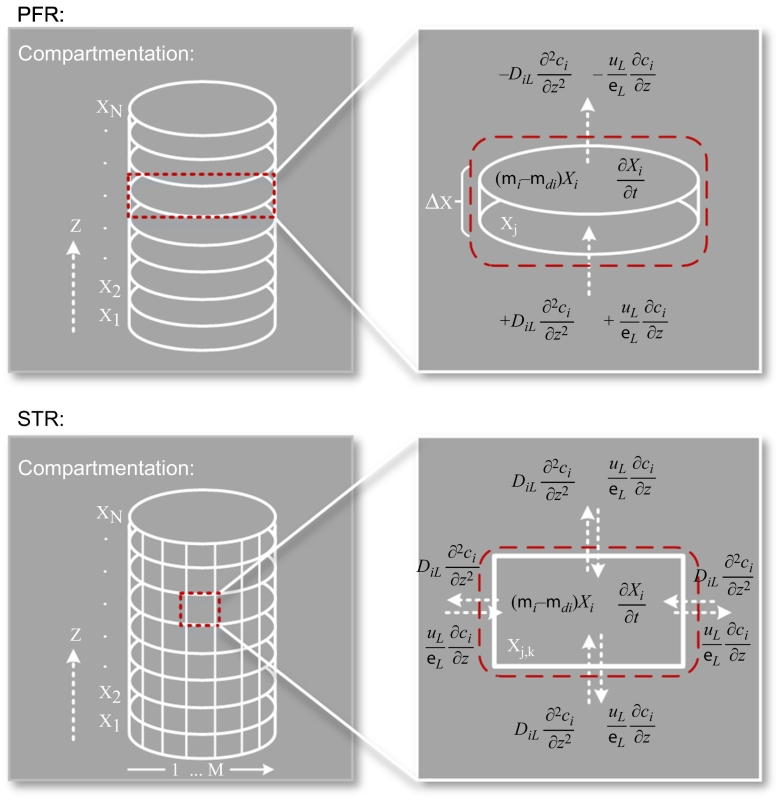
Compartmentation of a PFR and STR. Each section is homogeneously mixed and represented by a partial differential equation that usually has an accumulation term, a source/sink term due to bacterial growth and death, and convection and diffusion terms (in/out) which describe the environmental conditions. Other than the PFR, the STR needs finer discretization due to tangential mixing.

Another way to predict hydrodynamics is the use of compartment models (CM) [130]. Characteristically, the reactor is divided into a subset of spatial parts, each assumed to be ideally mixed, see Fig. 4.

Compartment models are much less computationally demanding than CFD simulations and moreover, allow easy implementation of complex reaction schemes. Fluxes between the compartments are often defined by considering global quantities which are not representative of the flow complexity. Moreover, incoming concentrations are instantly ideally mixed in the whole compartment and erratic changes occur, which are not observed in reality [24].

**Fig. 5:**
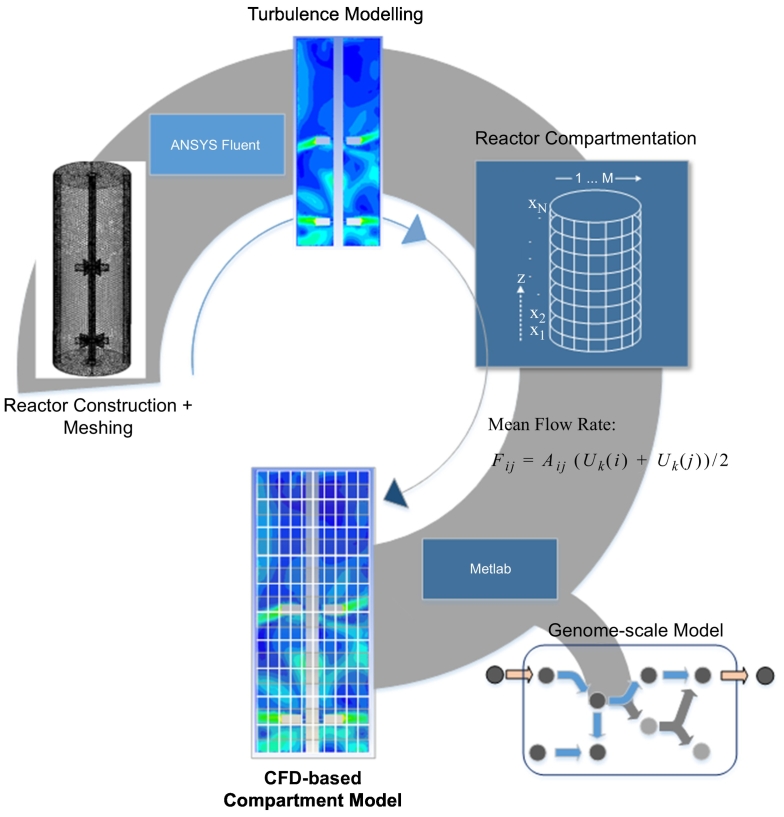
CFD-based compartment model set-up steps. (1) Construction and meshing of the reactor, (2) CFD simulation of the turbulent flow (velocity components, dissipation rate, turbulent kinetic energy), (3) Definition of reactor compartmentation, (4) Definition of mean flow F_*ij*_ between cell i and cell j, where U_*k*_(i) is the velocity component and A_*ij*_ is the area of the face between the two cells, (5) Incorporation of genome-scale model. Common simulation frameworks: Step (1) & (2): ANSYS Fluent, Step (3), (4) & (5): Matlab.

Recently, the combination of CFD and CM modeling has been presented to couple the accuracy of hydrodynamic CFD simulations with the simplicity and speed of compartmented modelings [24, [131], [132], [133]]. As shown in Fig. 5, the approach can be applied for describing concentration gradients in industrial-scale bioreactors, calculating the inter-compartmental fluxes from CFD velocity fields. Characteristically, turbulent liquid flows are computed by CFD first, followed by the implementation of net mean and turbulent flow rates in the compartment approach. The simplicity of the approach even allows complex genome-scale kinetic models to be used.

Likewise for gene regulatory models, fluid flow simulations must be validated based on independent experimental data. However, experimental observation of large-scale hydrodynamics is often lacking, which limits the comparison with the predicted flow patterns.

### 4.2 Hydrodynamic Modeling Linked to GRN Models

The physiological state of microorganisms and its impact on growth and product formation is the result of a complex interaction between the cellular environment and the cells. Large-scale studies have shown that homogeneous culture conditions are difficult to establish, nevertheless process engineering and bioreactor design may aim to create the least heterogeneous impact possible [134].

So far, large-scale simulations almost entirely focused on the integration of metabolism kinetics. They basically mirror the instantaneous cellular response on environmental changes [21, 143, 144]. However, cells react in a multi-response, multi-layer fashion also comprising the on- and offset of transcriptional regulation programs. Such responses are triggered in poorly mixed zones and are propagated into well mixed zones [14, 99]. Initiation and execution may be spatially disconnected which differs fundamentally from the metabolic responses studied so far.

To investigate the consequences of environmental heterogeneities, proper modeling frameworks should link local variations with cellular and subcellular kinetics.

The tool of choice is CFD simulation which can link the interaction of cellular activities with local environments [22, 135, 136].

Regarding the Euler-Euler approach, the liquid phase and the microorganisms are considered as a continuum. A continuum is a continuous system which does not allow erratic changes [137]. However, microorganisms are individual in their behavior and therefore the continuum description is a greatly simplified assumption. As a result, the continuum approach leads to a lack of individual responses of the cells.

Conventional Euler-Euler approaches of two-phase flow scenarios can be extended considering Population Balance Equations (PBEs) with unstructured kinetic growth models [[138], [139], [140], [141]]. PBEs are used to model population adaptation dynamics considering nutrient gradients inside large-scale bioreactors. In general, Euler-Euler approaches in combination with PBEs are suited to model particle (cell) swarms that follow flow patterns in the reactor [142].

However, an inherent limitation of PBEs is that the incorporation of a detailed kinetic network leads to massive computational effort because of high dimensional distribution functions that need to be solved. Additionally, no information on the level of single particles, such as their lifelines and history can be obtained with this approach.

This limitation can be tackled by using the Euler-Lagrange approach, which tracks the fate of each particle (cell) individually. The Lagrangian implementation requires detailed metabolic models of the cell, e.g. to describe the transport processes across the cellular membrane, via substrate uptake rates and product excretion rates. For simplification, massless cells are often used which are described via Monod-like black-box models. Such cells are assumed to travel along the flow fields thereby experiencing substrate gradients.

Notably, the cellular environment is typically ‘frozen’, i.e. fundamental cellular reactions are implemented in the Euler continuum and traveling cells only respond to the given hydrodynamic and concentration gradients [143]. Pioneering studies have been performed by Lapin et al. [18] and have been elaborated further in many follow-up studies [21, [143], [144], [145]].

Such studies clearly outline that cells are subject to repetitive and fast changes which in turn create heterogeneity within the population.

However, the computational effort for the spatial resolution of the conservation equations is high, requiring smart compositions of the computational grid and, for simplifying, assumptions to solve the numerical problem. Recently, Chen et al. [146] used a rather simple CM approach to simulate a syngas fermentation of *Clostridium ljungdahlii*. As a result, they could show that multi-compartment approaches, even if not widely used yet, give good results regarding the interaction of rather complex cells with their environment. Thus, in situations where a simple model structure meets the requirements of the modeling purpose, non-essential details should be avoided since they will unnecessarily prolong the modeling process.

## 5 Conclusion and Perspectives

Understanding the function of cellular behavior under varying conditions requires the development of computational approaches that incorporate gene regulatory models as well as environmental perturbation simulations relying on reliable experimental evidence.

On the one hand, due to efficient large-scale simulations and stimulus/response experiments, experimental findings have revealed a complex organization of regulatory response in the cell and improved the understanding of several regulatory processes. To further expand this understanding, development towards single cell resolution techniques has evolved. Although this is at the very beginning, this topic has significant potential for further developments regarding reactor design and genetic engineering towards robust strains.

On the other hand, numerical gene regulatory models based on ODE systems or modeling on a single molecular level with stochastic algorithms in combination with hydrodynamic simulations provided a broad and detailed insight into the regulatory mechanisms of microorganisms inside large-scale bioreactors. But due to a lack of large-scale experimental data, many regulation theories are still based to some extent on empirical observations. To date, hydrodynamic simulations as well as kinetic cellular models are available with different scales of complexity which favors the usability regarding the computational effort.

It could also be shown that a combination of already existing methods is often advantageous, such as CFD-based compartment models, providing the possibility of combining genome-scale models with hydrodynamic simulations.

Based on the extended variety and good results of cellular and hydrodynamic modeling approaches and the availability of reliable experimental data allowing detailed insight into cellular mechanisms, the time is right to use and combine these methods to predict the large-scale performance of microbial producers.

However, the above discussion has highlighted the need for knowledge-based process scale-up by elucidating the putative contributions of modeling. The contribution of numerical simulations also warrants further investigation with in vivo experiments that incorporate large-scale conditions and single cell resolution. The development towards automated high resolution processes and the detection of single cell behavior is a promising trend. This review shows that the basis to predict in silico large-scale performance of microbial producers is given. As a result, robust strains, as well as reactor design parameters and optimized cultivation conditions for more efficient processes can be developed.

## Conflict of Interest

None declared.
